# Event and Comment

**Published:** 1915-02

**Authors:** 


### DENTAL REGISTER

Vol. LXIX FEBRUARY 1915 No. 2

### EVENT AND COMMENT.

THE FOUR YEAR COURSE. The National Faculties Association, at its meeting just held, has decided to start a four-year course in Dentistry with the session beginning October 1, 1917. This Association has a membership of 41 schools and its action, with that taken by the Dental Faculties Association of American Universities to begin a four-year course in 1916, make a practically unanimous decision on this much-discussed question. The action taken by the same schools in 1902 and rescinded after only one year of trial was evidently premature. It is to be hoped that all schools will cordially support this movement and that it will become the standard of requirement the world over, as it now is in most countries. In 1902 only two years of high-school work were required for admission, whilst now four years and graduation is the requirement in most schools. With four years of preparatory high-school work and four years of professional training, we should educate better dentists than ever before. Some educators oppose the four-year curriculum because it deprives many men who would like to enter the profession from doing so, because of the expense involved. They claim that with the present high-school requirement for admission, and the three years of eight months in each session, that it costs a young man in actual outlay of money $2,000 to get a dental education. And if he waits until he has saved this money after leaving high school he can hardly hope to get into practice until he is 25 years old at best, and by this time he is getting beyond the period in life where he is likely to develop the delicate and skillful handicraft so essential in the practice of dentistry. This objecton, of course, has weight in their opinion and will be correspondingly increased by the four-year requirement. The argument is, however, not applicable to the majority of students who enter directly from the high school at the general age of 18 years, and some at 17 years; and these students as a rule have parents or friends who furnish the necessary funds. So that a very considerable number of students now graduate at 21 and 22 years of age, which is as early as such responsibilities as a profession like dentistry carries can be legally or morally placed upon men. The new course of four years is needed to permit the teaching of the constantly growing curriculum of the sciences and arts of our profession, and to meet the increasing demands for better dentistry by our patients. Colleges where emphasis has been placed largely on the so-called practical subjects of the curriculum do not feel the need of more time for teaching so much as in those schools where the fundamental sciences are considered as important as the more practical or applied branches of study. Any one who will compare the present requirements in the technical department of dentistry of today with the requirements in 1902, when a four-year course was first advocated, will easily see that tremendous advances have been made since then, and these advances in some cases require a deeper and closer study of the underlying sciences. Thirteen years ago we were not generally teaching modern orthodontia, porcelain and gold inlay methods, cast metal dentures, or oral hygiene and prophylaxis, to say nothing of modern methods of diagnosis with the X-rays, or of the newer methods of producing anesthesia for dental operations. A critical analysis of the situation will surely demonstrate that the profession has developed faster than our teaching methods and facilities. After, all the matter should be adjusted on the basis of our needs. Today the public is demanding a better educated dentist than we were producing a decade ago, and even if it does cost more to produce him, we can't help ourselves. Some schools will take the lead and get the credit for producing the kind of dentists the people need, and those schools who can't read correctly the signs of the times will be left behind. It is better that the action should be concerted, and we are glad that it has come unanimously and without legislative compulsion.

AMERICAN INSTITUTE OF DENTAL TEACHERS. The twenty-second annual meeting of this body was held in the Dental College of the University of Michigan, January 26th to 28th. Nearly 150 teachers from all parts of the United States and Canada were in attendance, and the proceedings were full of interest, and the greatest harmony and enthusiasm characterized the meeting. When this body was organized it was intended only to get together the instructors in Dental Anatomy as applied to operation technics. Its scope of work has now enlarged to the extent that every subject taught in the dental curriculum is constantly kept up to date by frequent considerations in its pedagogic and administrative relations. It would seem that the time must come when the interest in this organization would decrease, and that it would be difficult to present programmes that would claim the attention and interest of the older teachers. Many of those in attendance stated to us that they believed this meeting was the best one ever held, and it certainly was a most interesting meeting from many viewpoints. When such men as Drs. J. D. Patterson, Trueman W. Brophy, J. D. Willmot, S. H. Guilford, B. Holly Smith, D. M. Cattell, and Henry W. Morgan, think enough of this Association to leave their homes and travel long distances to attend and participate in its proceedings, there must be something worth while in it. They and many others find the proceeding so interesting and important that they keep coming year after year. Then there was Harry Carlton, one of the original organizers, who came all the way from San Francisco, and he is not now a teacher.

There were papers and discussions on such subjects as: The Making of the Dental Student a Better Student Dentist; Teaching of Prosthetic and Operation Dentistry; Teaching of Oral Hygiene; Operative Technics; Prosthetic Technics; and Dental Nomenclature. There were reports and discussions of various standing committees; exhibits of technical work by the various schools; textbooks by all the dental textbook publishers; and innumerable conferences of committees and individuals on the various ways and means of conducting classes, clinics and other forms of school activities. Three sessions were held each day, and the attendance was constant and everyone was alert for new and helpful information. A practitioner in attendance said to us that if that was a sample of this organization's meetings he was strongly inclined to enlist in the ranks of the pedagogues. The apparent spontaneity of these meetings is not altogether accidental as the Ad Interim Committee, or Executive Board, as it is officially called, has already begun the preparation for next year's meeting, which is to be held in Minneapolis. Before the present college sessions close the next year's programme will be largely known and the men who are to provide the papers and reports will be at work. This is a tradition of many years' standing which in large measure accounts for the value of the proceedings and the continued interest in this organization. This organization passes no legislation; in fact, it does not even require its members to endorse a code of ethics. It elects officers who appoint committees and plan its work from year to year. Dr. H. M. Semans, of the Ohio State University is President, and Dr. John F. Biddle has been the efficient Secretary for several years.

